# Feasibility and efficacy of simultaneous integrated boost intensity-modulated radiation therapy in patients with limited-disease small cell lung cancer

**DOI:** 10.1186/s13014-014-0280-9

**Published:** 2014-12-11

**Authors:** Dan Han, Qin Qin, Shaoyu Hao, Wei Huang, Yumei Wei, Zicheng Zhang, Zhongtang Wang, Baosheng Li

**Affiliations:** Department of 6th Radiation Oncology, Cancer Prevention and Treatment Institute of Shandong Province, Shandong, 250117 China; School of Medicine and Life Sciences, University of Jinan–Shandong Academy of Medical Sciences, Shandong, 250062 China; Department of Thoraic Surgery, Cancer Prevention and Treatment Institute of Shandong Province, Shandong, 250117 China

**Keywords:** Small-cell lung cancer, Lung neoplasms, Limited-stage, Simultaneous boost intensity-modulated radiotherapy, Outcome assessment

## Abstract

**Purpose:**

To evaluate the feasibility and efficacy of simultaneous integrated boost intensity-modulated radiation therapy (SIB-IMRT) in patients with limited-disease small-cell lung cancer (LD-SCLC).

**Methods:**

Patients with LD-SCLC were treated with SIB-IMRT within 1 week after completion of 2 cycles of induction chemotherapy. Then 2-4 cycles of adjuvant chemotherapy were administered within 1 week after SIB-IMRT. Irradiation was given accelerated hyper-fractionated with the prescribed dose 57Gy at 1.9Gy twice daily to the gross tumor volume (GTV) , 51Gy at 1.7Gy twice daily to the clinical tumor volume (CTV) and 45Gy at 1.5Gy twice daily to the planning target volume (PTV). The chemotherapy regimen consisted of platinum plus etoposide. Prophylactic cranial radiation (25Gy in 10 fractions) was administered to patients who got complete response (CR) or near complete response (nCR). The primary endpoint of this study was the frequency of grade 3 or higher acute non-hematologic treatment-related toxicities. Secondary end points included objective response, overall survival (OS), progression-free survival (PFS), locoregional recurrence-free survival (LRFS).

**Results:**

A cohort of 35 patients were enrolled in the study, the biological equivalent dose (BED) of the GTV in the SIB-IMRT was 59.16Gy. Grade 1, 2, and 3 esophagitis were observed in 11 (31%), 12 (34%), and 6 (17%) patients, respectively; Grade 1 and 2 pneumonitis were observed in 8 (23%) and 4 (11%) patients, respectively. The median OS and PFS of the whole group were 37.7 months and 29.3 months, respectively. The 1- and 2-year OS was 94.1% and 68.5%, respectively. The 1- and 2-year PFS was 76.8% and 40.7%, respectively. The 1- and 2-year LRFS was 87.7% and 73.8%, respectively.

**Conclusions:**

SIB-IMRT was feasible and well-tolerated in patients with LD-SCLC, and worth further evaluating in a large prospective clinical trial.

## Introduction

Small cell lung cancer (SCLC) accounts for 10%-15% of all lung cancer cases [1]. At the time of diagnosis, 30%-40% of SCLC patients present with limited disease (LD) which is defined as disease confined to the hemithorax and the regional lymphatic nodes according to the Veterans Administration Lung Study Group staging [2].

The combination of thoracic radiotherapy (TRT) with chemotherapy has been shown to be the standard treatment for limited-disease small-cell lung cancer (LD-SCLC) with improved local control and overall survivals based on two meta-analyses in the 1990s [3,4]. TRT with concurrent chemotherapy was endorsed by many studies because of better local control and longer survival although accompanied with higher toxicities compared with sequential chemoradiothearpy.

TRT is an indispensable treatment for LD-SCLC, however, the optimal radiotherapy approach remains controversial with respect to timing, dose-fractionation, and target definition. As far as dose-fractionation is concerned, accelerated hyper-fractionated radiotherapy (45Gy with 1.5Gy twice daily in 3 weeks) and dose-escalated conventional radiotherapy (60-70Gy with 2Gy once daily in 6 to 7 weeks ) have been documented as reliable schedules, and an international randomized trial is ongoing to compare these two schedules concurrent with chemotherapy in the treatment of LD-SCLC [5].

Despite of high sensibility to chemotherapy and radiotherapy, SCLC is characterized by inevitable local recurrence and distant metastasis due to its aggressive nature. As a dose-response relationship exists in treating LD-SCLC, it is reasonable to apply appropriately high doses to adequate target volumes as long as increased toxicities are acceptable to achieve better local control and subsequently longer survival [6]. Komaki et al. [7] found that high-dose thoracic radiation given twice daily during cisplatin-etoposide (EP) chemotherapy for LD-SCLC improved the rates of local control. Simultaneous integrated boost intensity-modulated radiotherapy (SIB-IMRT), in which different dose prescriptions can be delivered simultaneously to different target volumes in the same treatment fraction, has been advocated in recent years as a dose intensification technique, in which the overall treatment time was reduced, but boosted doses to corresponding volumes were produced [8-11].

To investigate the feasibility and effectiveness of SIB-IMRT as a dose intensification technique for patients with LD-SCLC, we conducted a single-center, open-label, and prospective phase II clinical study. The primary objectives of the study were to determine the safety and tolerability of SIB-IMRT in combination with chemotherapy. The secondary objectives included response rate, local control rate, and survival rate of SIB-IMRT in combination with chemotherapy.

## Methods and materials

### Eligibility criteria

The inclusion criteria included: Eastern Cooperative Oncology Group (ECOG) performance status of 0-2 grade; life expectancy ≥ 6 months; less than 75 years; no chemotherapy or radiotherapy prior to the study; no serious complications, such as hypertension, coronary heart disease, and psychiatric history; hemoglobin ≥ 100 g/L; white blood cell count ≥ 3.5 × 10^9^/L; neutrophil count ≥ 1.5 × 10^9^; platelet count > 100 × 10^9^/L; serum creatinine ≤ 1.5 × the upper limit of normal (ULN); serum bilirubin ≤ 2.5 × ULN; Glutamic oxalacetic transaminase and glutamic-pyruvic transaminase ≤ 2.5 × ULN; and alkline phosphatase ≤ 2.5 × ULN.

### Exclusion criteria

The exclusion criteria were as follows: known distant metastasis; history of carcinoma other than SCLC; other serious diseases such as myocardial infarction in the last 6 months; participation in other clinical trials in the last 4 weeks or at present; use of other anti-cancer drugs; and a history of organ transplantation. The study was performed after obtaining patients’ consent and under protocols approved by the institutional review boards of the Shandong Cancer Prevention and Treatment Research Ethics Committee.

### Pre-treatment evaluation

Patients were required to undergo a complete medical history before enrollment in the study. To exclude distant metastases, the pre-treatment assessment included a bone scan and computed tomography (CT) scan of the head, neck, chest, and abdomen. A physical examination, electrocardiogram, complete blood count, urinalysis, chemistry tests (including liver and kidney function tests), electrolytes, coagulation panel, and tumor markers were also required. Disease staging was performed according to the American Joint Committee on Cancer (AJCC version 7.0).

### Chemotherapy

The patients were treated with 2 cycles of induction chemotherapy, followed by TRT within 1 week after completion of the second cycle of chemotherapy. Then 2-4 cycles of adjuvant chemotherapy were administered within 1 week after completion of TRT. Chemotherapy consisted of etoposide (100 mg/m2 intravenously on days 1-5) and cisplatin (25 mg/m2 intravenously on days 1-3) and was administered every 3 weeks.

### Radiotherapy

The patients were immobilized using plastic mesh mask in the supine position and then, consecutively underwent four-dimensional computed tomography (4DCT) scanning under free breathing conditions on a 16-slice CT scanner (Philips Brilliance Bores CT). During the scanning procedure, respiratory signals were recorded using the Varian Real-Time Position Management respiratory gating hardware. After 4DCT scanning, the 4D software read all reconstructed images along with the respiratory phases. Images were evenly sorted into 10 phases distributed over a breathing cycle and all CT images were imported into the treatment planning system (Eclipse 8.6, Varian Medical Systems, Palo Alto, CA).

The targets were delineated according to the following criteria. Gross tumor volume (GTV) was manually delineated on all 10 phases of the 4DCT scan. The GTV referred to the restaging chest CT obtained after induction chemotherapy, including the residual primary tumor and all clinically-involved lymphatic regions. When enlarged lymph nodes resolved after induction chemotherapy, the previously involved lymph node regions were still included in the radiation target by reviewing the pre-chemotherapy CT scan. Elective treatment of clinically uninvolved lymphatic regions was not carried out. The clinical tumor volume (CTV) was defined by the expanding GTV with a 0.5 cm margin and the planning target volume (PTV) was defined by the expanding CTV with a 0.5 cm margin. The prescribed dose was 57Gy in 30 fractions at 1.9Gy per fraction to the GTV, 51Gy in 30 fractions at 1.7Gy per fraction to the CTV, and 45Gy in 30 fractions at 1.5Gy per fraction to the PTV.

All fractional doses were given twice daily with at least 6 h between fractions and 5 times each week. All plans aimed to cover at least 95% of the PTV with the 90% isodose, to have minimum dose > 90% and maximum dose < 110%. The dose–volume histogram (DVH) constraints of the organs at risk (OARs) were as follows: mean lung dose < 20Gy and lung V20 < 33%, spinal cord Dmax ≦ 45Gy, mean heart dose < 30Gy and heart V40 < 46% , mean esophagus dose < 34Gy, esophagus V35 < 50% [12,13].

The patients were treated based on a twice-daily three-dimensional cone beam computed tomography (CBCT) anatomy registration. CBCT images were acquired using a scanner attached to the gantry of the Trilogy Linear Accelerator (Varian Medical Systems). The discrepancy between planned and actual tumor position was automatically evaluated based on the automated alignment software. When the quality of a known parameter (such as bony landmarks in the chest) was ambiguous, the tumor contour was manually aligned to verify the automatic alignment results.

The biological equivalent dose (BED) was calculated using the linear quadratic formula: $\mathrm{BED}=\left(\mathrm{n}\mathrm{d}\right)\left[1+\mathrm{d}/\left(\alpha /\beta \right)\right] - \left(0.693t/\alpha \mathrm{Tpot}\right)$, where n = the total number of fractions delivered; d = the dose per fraction (Gy); α/β = 10 for acute effects and tumor control and 3 for chronic effects; α = 0.3Gy^-1^; t = total days in which radiotherapy is delivered; and Tpot = potential doubling time (5.6 days) [14].

### Prophylactic cranial irradiation (PCI)

After completion of chemotherapy and TRT, patients who achieved a complete response (CR) or near complete response (nCR) were offered the option of PCI. Patients were administered PCI (25Gy in 10 fractions to the entire brain) within 4 weeks after completion of all chemotherapy.

### Adverse effect assessment

Side effect assessment was graded at least weekly using the National Cancer Institute Common Toxicity Criteria (version 3.0) during radiotherapy and chemotherapy period. 3 months after the treatment, late toxicities were evaluated according to the Radiation Therapy Oncology Group (RTOG)/European Organization for Research and Treatment of Cancer late radiation morbidity scoring schema.

### Follow-up

The treatment response was estimated using CT or positron emission tomography/computed tomography after treatment, according to the Response Evaluation Criteria in Solid Tumors (version 1.0). Follow-up after treatment completion was every 3 months over the first 2 years and every 6 months thereafter. Each visit included medical history, physical examination, complete blood count, chest and abdomen CT, brain magnetic resonance imaging/CT, and bone scan (if necessary).

### Study endpoints and statistics

The primary endpoint of this study was the frequency of grade 3 or higher acute non-hematologic treatment-related toxicities. Secondary end points included objective response, overall survival (OS), progression-free survival (PFS), locoregional recurrence-free survival (LRFS). OS was observed from the first day of treatment until death or last follow-up time, PFS was observed from the first day of treatment until progress, death or last follow-up time, and LRFS was observed from the first day of treatment until recurrence, death or last follow-up time. The Kaplan–Meier method was used to estimate OS, PFS and LRFS using SPSS® v. 17.0 (SPSS Inc, Chicago, IL).

## Results

### Patient characteristics

Between AUG, 2009 and FEB, 2013, 44 SCLC patients in Shandong Cancer Hospital & Institute were enrolled in the study. 9 patients were excluded from the analysis of eligible patients, and leaving 35 assessable patients. The reasons for ineligibility were extensive disease (3), incomplete staging studies (1), absence of study data (2) and lose of follow up (3). All of the 35 patients finished the planned induction chemotherapy, radiotherapy, and adjuvant chemotherapy. 22 patients were given PCI within 4 weeks after completion of all chemotherapy. Of the 35 patients, 14 patients had stage IIIA, 15 had stage IIIB,4 had stage IIA, 1 had stage IIB, and 1 had stage IA. The median age was 54 years (range, 35-72 years). The patients’ characteristics were summarized in Table 1.

**Table 1:** **Characteristics of 35 patients**

**Characteristic**	**Number of cases (%)**
Age (y)	54 (35-72)
Gender	
Male	26 (74%)
Female	9 (26%)
ECGO performance status	
0	12 (34%)
1	22 (63%)
2	1 (3%)
T-stage (AJCC 7)	
1	4 (11%)
2	9 (26%)
3	10 (29%)
4	12 (34%)
N-stage (AJCC 7)	
0	3 (9%)
1	4 (11%)
2	23 (66%)
3	5 (14%)

Abbreviations: *ECOG* Eastern Cooperative Oncology Group.

*AJCC* American Joint Committee on Cancer, *T* Tumor, *N* Node.

### Radiotherapy plan evaluation

The BED of the GTV in the SIB-IMRT was 59.16Gy. The evaluation of the DVH-based parameters of the OARs were shown in Table 2.

**Table 2:** **The DVH-based parameters of the OARs in the study**

	**mean ± SD**
**Total lungs**	
MLD (Gy)^a^	14.8 ± 1.7
V5 (%)^b^	67.4 ± 9.0
V10 (%)^b^	53.5 ± 7.2
V20 (%)^b^	28.7 ± 3.2
V30 (%)^b^	17.1 ± 2.6
**Ipsilateral lungs**	
MLD (Gy)	22.1 ± 4.1
V5 (%)	79.1 ± 10.7
V10 (%)	70.7 ± 11.8
V20 (%)	47.2 ± 11.3
V30 (%)	30.3 ± 10.0
**Contralateral lungs**	
MLD (Gy)	8.8 ± 2.9
V5 (%)	53.1 ± 13.1
V10 (%)	32.1 ± 12.7
V20 (%)	11.8 ± 8.8
V30 (%)	5.2 ± 4.6
**Spinal cord**	
Dmax (Gy)^e^	42.7 ± 3.7
**Heart**	
Dmean (Gy)^a^	16.1 ± 7.3
V30 (%)^c^	23.3 ± 15.0
V40 (%)^c^	9.0 ± 5.0
**Esophagus**	
MED (Gy)^a^	29.0 ± 6.7
V45 (%)^d^	33.6 ± 5.3

Abbreviations: *SD standard deviation.* ^a^The mean irradiation dose that the lung, heart and esophagus received, respectively; ^b^the volume of the lung that received the 5, 10, 20 and 30Gy irradiation dose, respectively; ^c^the volume of the heart that received 30 and 40Gy irradiation dose, respectively; ^d^the volume of the esophagus that received 45Gy irradiation dose; ^e^the maximum irradiation dose that spinal cord received.

### Toxicity

The toxicities for each patient were presented in detail (Table 3). 15(43%)and 7(20%)patients had grade 3 and 4 hematologic toxicity, respectively. Necessary treatment measures, such as recombinant human interleukin and granulocyte colony stimulating factor, were provided and blood transfusion were given to the patients with grade 4 hemoglobin toxicity (All patients fully recovered from hematologic toxicity). Of the 35 patients, grade 1, 2, and 3 esophagitis occurred in 11 (31%), 12 (34%), and 6 (17%) patients, respectively; the patients with grade 3 esophagitis required intravenous nutrition. Grade 1 and 2 pneumonitis occurred in 8 (23%) and 4 (11%) patients, respectively. None of the patients died of treatment-related causes.

**Table 3:** **Treatment-related toxicity**

	**Grade (n = 35) number (percent) of patients**			
**Category**	**1**	**2**	**3**	**4**
Hematologic toxicity (WBC)	4(11%)	21(60%)	9(26%)	1(3%)
Hematologic toxicity (PLT)	4(11%)	2(6%)	2(6%)	3(9%)
Hematologic toxicity (HB)	8(23%)	5(14%)	4(11%)	3(9%)
Stomach/intestine	13(37%)	11(31%)	2(6%)	0
Esophagitis	11(31%)	12(34%)	6(17%)	0
Pneumonitis	8(23%)	4(11%)	0	0
Weight loss	3(9%)	1(3%)	0	0
Skin (within the irradiated field)	2(6%)	0	0	0
Fever	1(3%)	3(9%)	0	0

Abbreviations: *WBC* white blood cell, *PLT* platelet, *HB* hemoglobin.

### Treatment response

The efficacy of induction chemotherapy was observed in 35 patients; 29 patients achieved partial response (PR), 4 patients achieved CR and 2 patients had stable disease (SD). 4 weeks after radiotherapy and chemotherapy, of the 35 patients, 51% of patients (n = 18) had CR, 11% of patients (n = 4) had nCR, and another 26% (n = 9) had PR, for an overall response rate of 88%; another 9% of patients (n = 3) had SD, 3% (n = 1) had progressive disease (PD), and no one died before the 4 weeks post-treatment response could be evaluated.

### Survival

**Figure 1:**
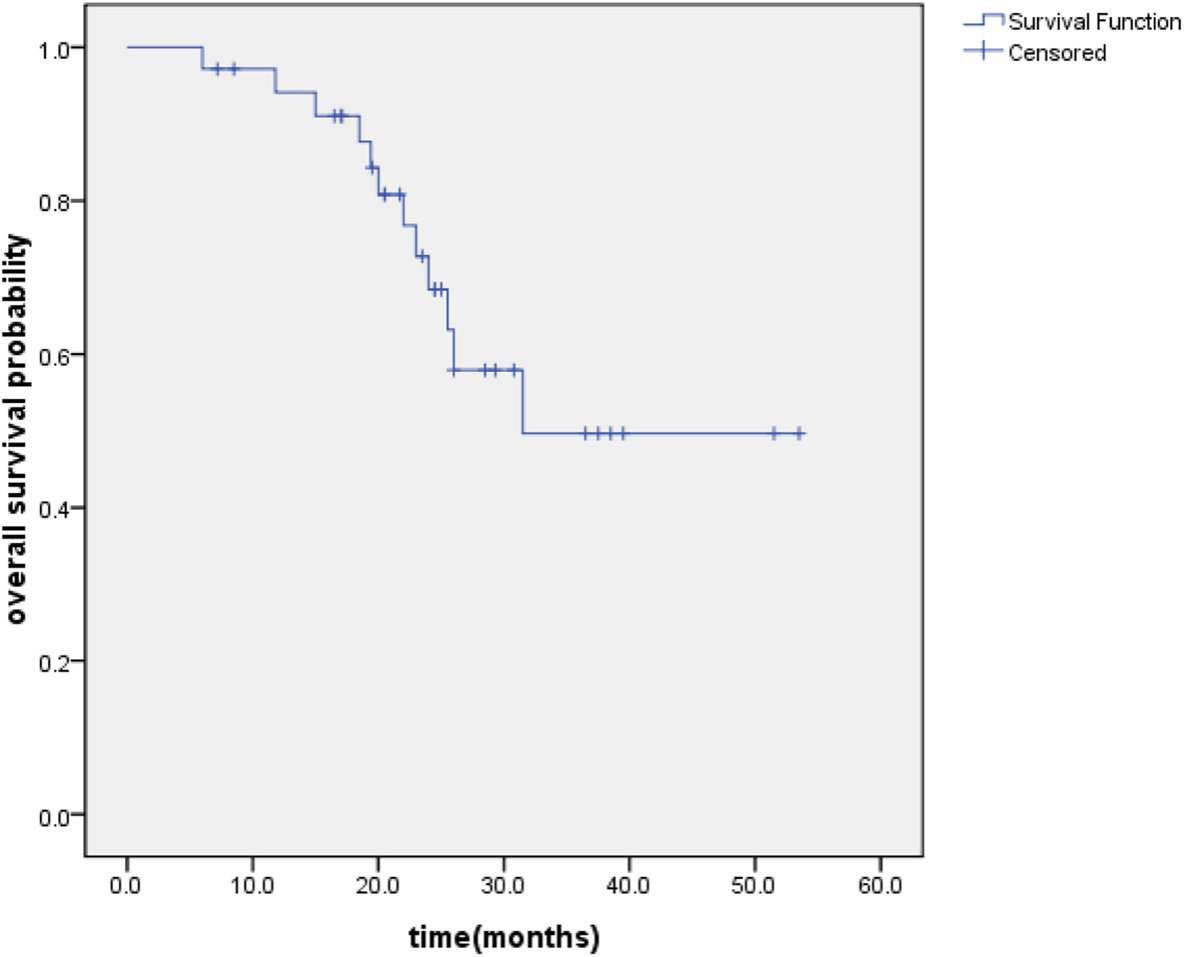
**Kaplan–Meier plot of overall survival.**

**Figure 2:**
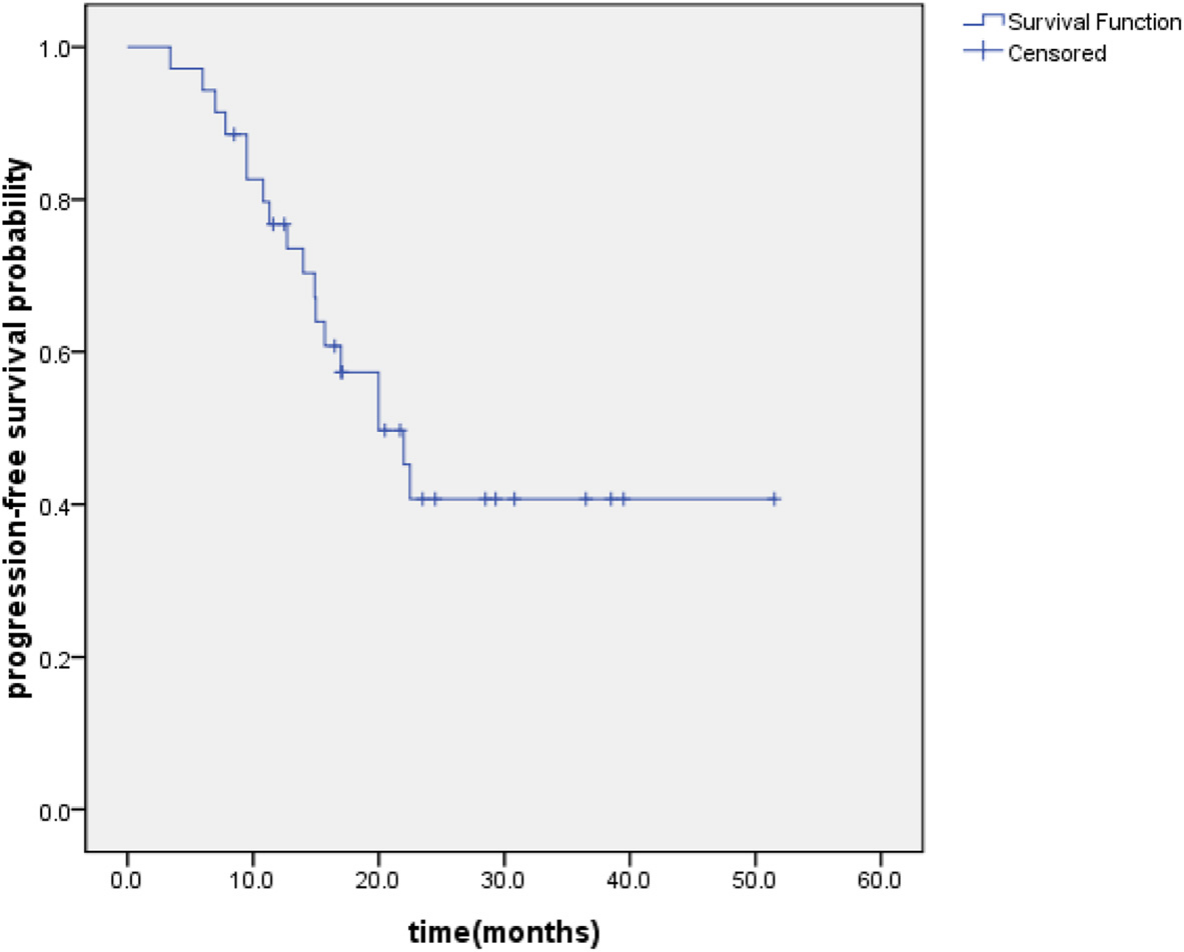
**Kaplan–Meier plot of progression-free survival.**

**Figure 3:**
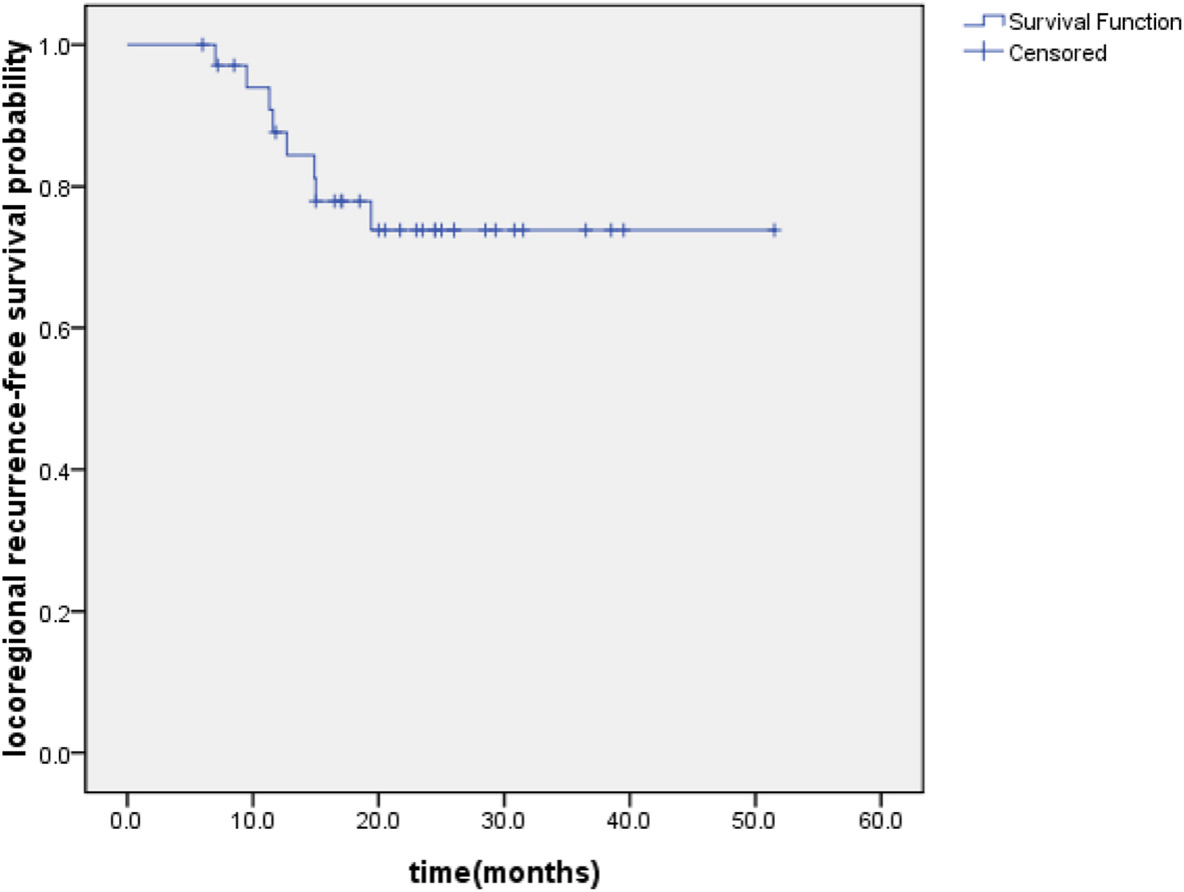
**Kaplan–Meier plot of locoregional recurrence-free survival.**

The median follow-up was 24.6 months, with a range of 6–53.5 months until the last follow-up date (15 August 2014). The median OS and PFS of the entire group were 37.7 and 29.3 months, respectively. The OS, PFS and LRFS were illustrated in Figures 1, 2, and 3, respectively. The 1- and 2-year OS were 94.1% and 68.5%, respectively. The 1- and 2-year PFS were 76.8% and 40.7%, respectively. The 1- and 2-year LRFS were 87.7% and 73.8%, respectively. At the last follow-up, 15 (43%) patients were alive with no evidence of failure. Local regional recurrences only developed in 4 (11%) patients and distant metastases only occurred in 12 (34%) patients. 4 (11%) patients had a local recurrence and distant metastases simultaneously. Among the 16 patients who developed distant metastases, the most common sites were brain (n = 6), followed by liver (n = 3), adrenals (n = 2), lung (n = 2), bone (n = 1), subcutaneous tissue (n = 1) and supraclavicular lymph nodes (n = 1).

## Discussion

The best method of integrating thoracic radiation with chemotherapy includes conflicting reports. Turrisi et al. [15] conducted a randomized trial that demonstrated a twice daily regimen of 45Gy/30 fractions over 3 weeks that produced an acute grade 3 esophagitis rate of 27%, a local regional failure rate of 36%, and a 2-year OS rate of 47% was superior to 45Gy in 25 daily fractions. The NCCTG 95-20-53 trial [16], which included 6 cycles of EP and cycles 4 and 5 included concurrent chemotherapy and TRT (30Gy/20 twice daily fractions, a 2-week break, and another 30Gy/20 twice daily fractions), resulted in a favorable 5-year survival rate of 24%; however, the locoregional failure remained a problem and grade 3 or grade 3+ toxicity were as high as 97%. In the RTOG 0239 study [7], patients with LD-SCLC were given thoracic radiation to 61.2Gy over 5 weeks (daily 1.8Gy fractions on days 1-22, then twice daily 1.8Gy fractions on days 23-33), and the rate of grade 3 esophagitis was 18% and local regional failure rate was 20%, but the 2-year OS rate of 36.6% did not reach the projected goal. A newer method should allow the safe administration of higher doses of TRT.

In the present study, we first applied SIB-IMRT that delivered GTV at 57Gy in 30 fractions, CTV at 51Gy in 30 fractions, and PTV at 45Gy in 30 fractions twice daily over 3 weeks. The dose could be escalated in a single plan for the entire treatment, but still met dose constraints to critical normal structures, such as the heart, lung, esophagus, and spinal cord. Favorable results were achieved, with grade 3 esophagitis rate of 17%, and 2-year OS of 68.5%, and 2-year LRFS was 73.8%. Of note, the sample size of the study was small.

Despite concurrent chemoradiotherapy represents the standard treatment for patients with LD-SCLC, we applied sequential chemoradiotherapy in the current trial considering serious toxicity from concurrent chemoradiotherapy and insufficient supportive treatment in developing country [17]; meanwhile, we supposed that sequential chemoradiotherapy may not be inferior to concurrent chemoradiotherapy. SCLC is characterized by the predominantly central type and bulky tumor [1], so hematologic toxicity and esophagitis are more severe in the concurrent regimen. Choi et al. [18] reported that esophagitis limited treatment when total dose from twice daily treatment exceeded 45Gy concurrent chemotherapy. Furthermore, in a phase III study from the Japanese Clinical Oncology Group , 231 patients who received 4 cycles of cisplatin plus etoposide were randomly assigned to either sequential or concurrent twice daily 45Gy TRT. There was no statistically significant difference (*p* = 0.097) in survival between the two groups [19].

Our results in the 35 patients treated according to the upper approach indicate clear feasibility. The main toxicity problems of our study were grade 3 esophagitis and grade 2 pneumonitis, affecting 17% and 11%, respectively. The 17% severe acute esophagitis rate in this study was higher than the 4% experienced in the sequential arm of Japanese Clinical Oncology Group Study 9104 because of the higher radiation dose [19]. Esophagitis after SIB-IMRT did not lead to limitation, and all the affected patients recovered their ability to swallow. The grade 3 esophagitis rate was lower than the 27% occurred in the twice daily arm of INT 0096. The INT 0096 protocol called for starting thoracic radiation on day 1 of chemotherapy on the basis of other studies showing that local control and survival were better when the radiation was started early relative to the chemotherapy [20,21]. The TRT in this study began within 1 week after completion of the 2^nd^ cycle of chemotherapy, which may allow us to reduce the volume of esophagus within the radiation fields and synergetic toxicity during therapy.

Thoracic RT affects the patient outcome by decreasing the tumor burden within the chest, resulting in enhanced local control and survival [3,4]. Despite the addition of thoracic RT to chemotherapy, local treatment failures occur in approximately one third of patients treated with the currently accepted optimal therapy in LD-SCLC [16]. Recent evidence supports the hypothesis that TRT dose intensification improves LD-SCLC patients’ outcomes [6]. Our results supported this view and showed a favourable outcome that the 2-year LRFS was 73.8%, which was in agreement with the data reported by RTOGP0239 [7].

As a dose-response relationship exists in treating LD-SCLC, local control and subsequent survival are associated with dose-fractionation parameters [13]. The 2-year OS rate of 68.5% in the present study was higher than the 47% in the twice-daily arm of INT 0096 and the 36.6% in the RTOGP0239. Compared to the INT 0096, we got a higher BED of 59.16Gy to the tumor with the SIB-IMRT. The BED can be used to compare the efficacy of various dose-fractionation regimens in providing tumor control and survival [22,23].

Distant metastasis was the dominant cause of failure and the most common site was brain (17%) in present study. Several meta-analyses and randomized trials have focused on the role of PCI in patients with SCLC. A meta-analysis [24] involving 987 patients with SCLC and a CR to chemotherapy showed a 5.4% increase in the 3-year survival for those undergoing PCI. Although Tai et al. [25] reported that patients with a PR benefited from PCI, with a reduced rate and delayed time for development of brain metastases, patients who got CR or nCR were given PCI in this study. 22 patients were given PCI and there were 10 patients in whom disease progressed after PCI (3 [14%] with brain metastases), which was higher than Tai et al. reported (9%).

In conclusion, the regimen was safe and well-tolerated, and demonstrated an encouraging outcome in patients with LD-SCLC. But this study based on small sample size and further randomized studies should be carried out.

## Abbreviations

SCLC: Small cell lung cancer
LD: Limited disease
TRT: Thoracic radiotherapy
LD-SCLC: Limited disease small cell lung cancer
EP: Cisplatin-etoposide
SIB-IMRT: Simultaneous integrated boost intensity-modulated radiotherapy
ECOG: Eastern cooperative oncology group
ULN: Upper limit of normal
CT: Computed tomography
AJCC: American joint committee on cancer
4DCT: Four-dimensional computed tomography
GTV: Gross tumor volume
CTV: Clinical tumor volume
PTV: Planning target volume
DVH: Dose volume histogram
OARs: Organs at risk
CBCT: Cone beam computed tomography
BED: Biological equivalent dose
PCI: Prophylactic cranial irradiation
CR: Complete response
nCR: Near complete response
OS: Overall survival
PFS: Progression-free survival
LRFS: Locoregional recurrence-free survival
PR: Partial response
SD: Stable disease
PD: Progressive disease

## References

[1] van Meerbeeck JP, Fennell DA, De Ruysscher DK (2011). Small-cell lung cancer. Lancet.

[2] Jemal A, Siegel R, Ward E, Hao Y, Xu J, Murray T, Thun MJ (2008). Cancer statistics 2008. CA Cancer J Clin.

[3] Pignon JP, Arriagada R, Ihde DC, Johnson DH, Perry MC, Souhami RL, Brodin O, Joss RA, Kies MS, Lebeau B (1992). A meta-analysis of thoracic radiotherapy for small-cell lung cancer. N Engl J Med.

[4] Warde P, Payne D (1992). Does thoracic irradiation improve survival and local control in limited-stage small-cell carcinoma of the lung? A meta-analysis. J Clin Oncol.

[5] Bogart J, Masters G, Komaki R, Heymach J (2008). Phase III Comparison of Thoracic Radiotherapy Regimens in Patients With Limited Small Cell Lung Cancer Also Receiving Cisplatin and Etoposide. Current Controlled Trials.

[6] Yee D, Hanson J, Butts C, Reiman A, Joy A, Smylie M, Fenton D, Chu Q, Roa W (2010). Phase I dose escalation trial of hypofractionated limited-field external beam thoracic radiotherapy for limited-stage small cell carcinoma of the lung. Radiother Oncol.

[7] Komaki R, Paulus R, Ettinger DS, Videtic GM, Bradley JD, Glisson BS, Langer CJ, Sause WT, Curran WJ, Choy H (2012). Phase II study of accelerated high-dose radiotherapy with concurrent chemotherapy for patients with limited small-cell lung cancer: radiation therapy oncology group protocol 0239. Int J Radiat Oncol Biol Phys.

[8] Franceschini D, Paiar F, Meattini I, Agresti B, Pasquetti EM, Greto D, Bonomo P, Marrazzo L, Casati M, Livi L, Biti G (2013). Simultaneous integrated boost–intensity‐modulated radiotherapy in head and neck cancer. Laryngoscope.

[9] Wu B, McNutt T, Zahurak M, Simari P, Pang D, Taylor R, Sanguineti G (2012). Fully automated simultaneous integrated boosted–intensity modulated radiation therapy treatment planning is feasible for head-and-neck cancer: a prospective clinical study. Int J Radiat Oncol Biol Phys.

[10] Deenen MJ, Dewit L, Boot H, Beijnen JH, Schellens JH, Cats A (2013). Simultaneous integrated boost–intensity modulated radiation therapy with concomitant capecitabine and mitomycin C for locally advanced anal carcinoma: a phase 1 study. Int J Radiat Oncol Biol Phys.

[11] Fogliata A, Bolsi A, Cozzi L, Bernier J (2003). Comparative dosimetric evaluation of the simultaneous integrated boost with photon intensity modulation in head and neck cancer patients. Radiother Oncol.

[12] Marks LB, Yorke ED, Jackson A, Ten Haken RK, Constine LS, Eisbruch A, Bentzen SM, Nam J, Deasy JO (2010). Use of normal tissue complication probability models. Int J Radiat Oncol Biol Phys.

[13] Gagliardi G, Constine LS, Moiseenko V, Correa C, Pierce LJ, Allen AM, Marks LB (2010). Radiation dose-volume effects in the heart. Int J Radiat Oncol Biol Phys.

[14] Liu HH, Wang X, Dong L, Wu Q, Liao Z, Stevens CW, Guerrero TM, Komaki R, Cox JD, Mohan R (2004). Feasibility of sparing lung and other thoracic structures with intensity- modulated radiotherapy for non-small cell lung cancer. Int J Radiat Oncol Biol Phys.

[15] Turrisi AT, Kim K, Blum R, Sause WT, Livingston RB, Komaki R, Wagner H, Aisner S, Johnson DH (1999). Twice-daily compared with once-daily thoracic radiotherapy in limited small-cell lung cancer treated concurrently with cisplatin and etoposide. N Engl J Med.

[16] Schild SE, Bonner JA, Hillman S, Kozelsky TF, Vigliotti AP, Marks RS, Graham DL, Soori GS, Kugler JW, Tenglin RC, Wender DB, Adjei A (2007). Results of a phase II study of high-dose thoracic radiation therapy with concurrent cisplatin and etoposide in limited stage small cell lung cancer (NCCTG 95-20-53). J Clin Oncol.

[17] Xia B, Chen GY, Cai XW, Zhao JD, Yang HJ, Fan M, Zhao KL, Fu XL (2011). The effect of bioequivalent radiation dose on survival of patients with limited-stage small-cell lung cancer. Radiat Oncol.

[18] Choi NC, Herndon JE, Rosenman J, Carey RW, Chung CT, Bernard S, Leone L, Seagren S, Green M (1998). Phase I study to determine the maximum-tolerated dose of radiation in standard daily and hyperfractionated-accelerated twice-daily radiation schedules with concurrent chemotherapy for limited-stage small-cell lung cancer. J Clin Oncol.

[19] Minoru T, Masahiro F, Masaaki K, Takahiko S, Akira Y, Soichiro Y, Yutaka N, Koshiro W, Kazumasa N, Tomohide T, Haruhiko F, Nagahiro S (2002). Phase III study of concurrent versus sequential thoracic radiotherapy in combination with cisplatin and etoposide for limited-stage small-cell lung cancer: results of the Japan Clinical Oncology Group Study 9104. J Clin Oncol.

[20] Murray N, Coy P, Pater JL, Hodson I, Arnold A, Zee BC, Payne D, Kostashuk EC, Evans WK, Dixon P (1993). Importance of timing for thoracic irradiation in the combined modality treatment of limited-stage small-cell lung cancer. The National Cancer Institute of Canada Clinical Trials Group. J Clin Oncol.

[21] De Ruysscher D, Pijls-Johannesma M, Bentzen SM, Minken A, Wanders R, Lutgens L, Hochstenbag M, Boersma L, Wouters B, Lammering G, Vansteenkiste J, Lambin P (2006). Time between the first day of chemotherapy and the last day of chest radiation is the most important predictor of survival in limited-disease small-cell lung cancer. J Clin Oncol.

[22] Fowler JF (2001). Biological factors influencing optimum fractionation in radiation therapy. Acta Oncol.

[23] Fowler JF (1989). The linear-quadratic formula and progress in fractionated radiotherapy. Br J Radiol.

[24] Aupérin A, Arriagada R, Pignon JP, Le Péchoux C, Gregor A, Stephens RJ, Kristjansen PE, Johnson BE, Ueoka H, Wagner H, Aisner J (1999). Prophylactic cranial irradiation for patients with small-cell lung cancer in complete remission. Prophylactic Cranial Irradiation Overview Collaborative Group. N Engl J Med.

[25] Tai P, Assouline A, Joseph K, Stitt L, Yu E (2013). Prophylactic cranial irradiation for patients with limited-stage small-cell lung cancer with response to chemoradiation. Clin Lung Cancer.

